# Application of 3D Printing in Implantable Medical Devices

**DOI:** 10.1155/2021/6653967

**Published:** 2021-01-12

**Authors:** Zhenzhen Wang, Yan Yang

**Affiliations:** College of Mechanical Engineering, Chongqing University of Technology, Chongqing, China

## Abstract

3D printing technology is widely used in the field of implantable medical device in recent decades because of its advantages in high precision, complex structure, and high material utilization. Based on the characteristics of 3D printing technology, this paper reviews the manufacturing process, materials, and some typical products of 3D printing implantable medical devices and analyzes and summarizes the development trend of 3D printed implantable medical devices.

## 1. Introduction

With the continuous improvement of people's health awareness, implantable medical devices to improve patients' life quality have also been widely used [1]. When the shape of the implantable medical devices is complex, it is often difficult to process and even unable to process. 3D printing technology can produce implantable medical devices with any complex shape, without having to consider processing problems, and solve the design and manufacturing problems of complex implantable medical devices. 3D printing technology plays an increasingly important role in implantable medical devices by virtue of its ability to print and shape most materials [2], its precision, personalized and other customized requirements [3], and its high material utilization rate in the printing process. This article briefly describes the application of 3D printing in implantable medical devices.

## 2. 3D Printing Manufacturing Process

3D printing is also known as additive manufacturing; it is done through layer-by-layer stacking techniques, and according to the designed 3D model, complex and diverse physical entities can be manufactured [4]. Common manufacturing processes for 3D printing include Stereo Lithography Apparatus (SLA) [5], Laminated Object Manufacturing (LOM) [6], Selective laser Sintering (SLS) [7], Fused Deposition Modeling (FDM) [8], and Three-Dimensional Printing (3DP) [9].

### 2.1. Stereo Lithography Apparatus

**Figure 1:**
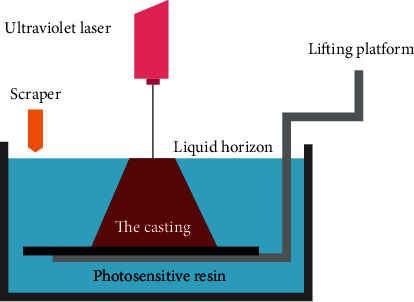
The working principle of Stereo Lithography Apparatus [10].

Stereo Lithography Apparatus is the earliest practical rapid prototyping technology in 3D printing technology. As early as 1984, it was proposed by Charles W. Hull in the United States and patented in 1986. In 1986, Charles W. Hull established 3D systems in and released the world's first commercial 3D printer SLA-250 in 1988. The working principle of Stereo Lithography Apparatus (Figure 1): a layer of powder material is laid flat on the upper surface of the formed part, then heated to a temperature exactly below the unsintered point of the powder. The control system controls the laser beam scanning on the powder layer in order the cross-section outline of the layer, making the powder temperature rise to the melting point, sintering and bonding with the formed part below. After the first layer is completed, the workbench lowers the thickness of one layer, spreads a layer of uniform and dense powder on it, and sinters the section of the new layer until the entire model is completed. Its basic composition is shown in Figure 1, including ultraviolet laser, lifting platform, scraper, liquid horizon, and photosensitive resin. This technology is widely used in orthopedic repair and tissue engineering and can be used to print skull and hip bones.

Stereo Lithography Apparatus has high molding efficiency and stable operation; the printed parts have high precision and high finish. But the equipment is relatively large, and there are still problems in the size of optical pixels, which limits the microstructure in the plane [10].

Due to the low penetration depth of ultraviolet light, the application of SLA technology exited by ultraviolet light sources is limited. So, a novel and cost-effective 3D printing technology has emerged: NaYF4:Yb3+/Tm3+ up-conversion microcrystals to produce internal deep ultraviolet (UV) light centers as a light curing source in the polymer matrix by near infrared light excitation. Excellent up-conversion emission from NaYF4:Yb3+/Tm3+ microcrystals is demonstrated by using microcrystals as gain medium to realize up-conversion lasing emission centered at 291,346 and 364 nm. This 3D printing technique, in which a low cost semiconductor laser is used, is potential to print large structures at high printing efficiency.

### 2.2. Laminated Object Manufacturing

**Figure 2:**
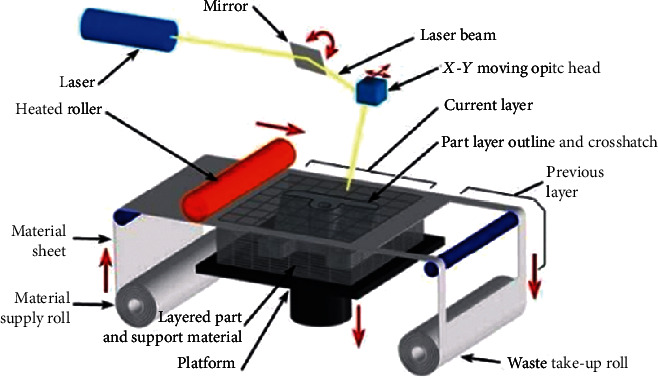
Laminated Object Manufacturing.

Laminated Object Manufacturing was first developed by the Helisys Company in 1986 and has been developed rapidly since it came out in 1991. Laminated Object Manufacturing is one of the most mature 3D printing technologies. The working principle of Laminated Object Manufacturing (Figure 2): Laminated Object Manufacturing (LOM) uses thin materials such as paper and plastic film. The surface of the sheet is coated with a layer of hot melt adhesive in advance. In the process of machining parts, the sheet is hot-pressed and radiated to bond it with the formed workpiece below is the first step, with CO_2_ laser on the new layer of adhesive cut out parts of section contour and the workpiece frame, and the cross section profile between the frame and the excess of aligned to carve up and down in the area of the grid; after the laser cutting is completed, the worktable drives the formed workpiece down and separates from the strip-shaped sheet (material belt); then, the feeding mechanism rotates the receiving shaft and the feeding shaft, to drive the material belt to move the new layer to the processing area, and the worktable rises to the processing plane; finally, the hot-pressing rod is used for hot-pressing; the number of layers of the workpiece is increased by one layer, so the height of the workpiece is increased by one layer, and then, the cross-section profile is cut on the new layer. This is repeated until all the cross-sections of the part are bonded and cut, and a layered manufacturing solid part is obtained. The general process of layered solid manufacturing technology molding is roughly graphics processing, substrate production, prototype production, residual material removal, and postprocessing. LOM including laser, heated roller, material supply roll, layered part and support material, platform, waste take-up roll, and part layer outline and crosshatch. The technique can be applied to orthopedics such as temporal bone, dental jaw, and mandible.

Since the materials processed by the layered solid molding process are easy to obtain, the raw material cost of the process is low, and the precision of the manufactured workpieces is relatively ideal, which is suitable for the molding of large-sized workpieces. However, the mechanical properties of the workpiece produced by this technology are relatively low, and compared with other printing technologies, the efficiency of the layered solid molding process is poor [11].

### 2.3. Selective Laser Sintering

**Figure 3:**
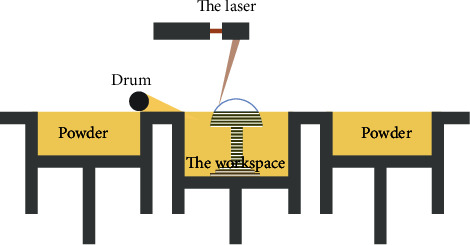
Working principle of selective laser sintering process.

The selective laser sintering process was first developed by DTM in 1987 and was patented in 1988 by C.R. Dechard of the University of Texas at Austin. In 1992, C.R. Dechard released sinter station, a commercial 3D printer based on selective laser sintering technology in the DTM Company, which was founded by C.R. Dechard. The selective laser sintering usual laser utilized is CO_2_ due to the absorption by the polymers being much more efficient at this wavelength. Working principle of selective laser sintering process (Figure 3): in a closed molding chamber install two cylinder piston mechanism, one of the cylinders for powder, the other for molding. Before the forming process begins, the powder material is heated to a temperature just below the sintering point using an infrared plate. At the beginning of forming, the piston in the powder-feeding cylinder moves up for a certain amount, the powder laying drum evenly spreads the powder on the processing surface of the forming cylinder, and the laser beam scans the first layer of information at a given speed and energy under the control of the computer. Where the laser beam sweeps the powder is sintered and solidified into a sheet of a given thickness. The unsintered powder is used as a support so that the first layer of the part is made. At this time, as soon as the molding cylinder piston moves down, the feed rainbow piston moves up, and the powder is laid and rolled again; then, the laser beam is scanned according to the second layer of information, and the second layer formed is also sintered and solidified on the first layer. In this way, a 3D solid part is produced by stacking layer by layer [12]. This process is basically the same as the Stereo Lithography Apparatus (SLA), except the SLA's liquid resin is replaced with a powder material that can be sintered under laser light; the roller is optimized by a temperature control unit to smooth the material to ensure the fluidity of the powder, and the heat of the working chamber is controlled to make the powder bond firmly. By melting the polymer in the powder, the metal powder with high melting point is bonded together. After forming, the binder is removed by degreasing process, leaving a large number of honeycomb holes in the material, which is extremely conducive to the preparation of porous materials. This technology can be used to make scaffolds to promote bone regeneration.

The most prominent advantages of selective laser sintering are the variety of materials that can be processed and the short production cycle. The disadvantages of this technology are the low strength and poor surface quality of manufactured parts [13].

### 2.4. Fused Deposition Modeling

**Figure 4:**
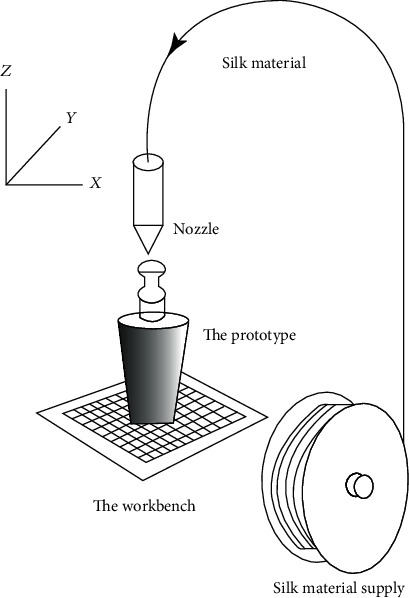
The working principle of fused deposition manufacturing [14].

Fused deposition manufacturing was first invented by Scott Crump in 1988. In 1992, Stratasys was granted a 3D printing technology patent for fused deposition manufacturing technology. In the same year, Stratasys launched the world's first 3D printer based on fused deposition manufacturing technology—“3D Modeler.” Fused deposition manufacturing is mainly used to mold thermoplastic polymer materials. The main principle (Figure 4) is the thermoplastic polymer wire is transported to a heating extruder via a feed unit, under the action of the heater above the nozzle based on the set process temperature, after heating to molten state, uniform extrusion, layer by layer on the forming platform and cooling solidification. When one layer of cross-section is completed, the worktable descends a certain height, and then, another layer is machined on the formed section, so as to repeat until the workpiece is manufactured [14], particularly widely used in bone engineering.

All of the fused deposition manufacturing technology can be widely used; the first of reasons is the fused deposition manufacturing technology does not use a laser, so compared with the laser-containing molding technology, its manufacturing cost is low. It can be machined with a variety of materials, high material utilization, to a large extent to reduce the cost of forming. However, the forming rate of this technology is low, and the surface of the workpiece has stripe sense, and the manufacturing precision is low [15].

### 2.5. Three-Dimensional Printing

**Figure 5:**
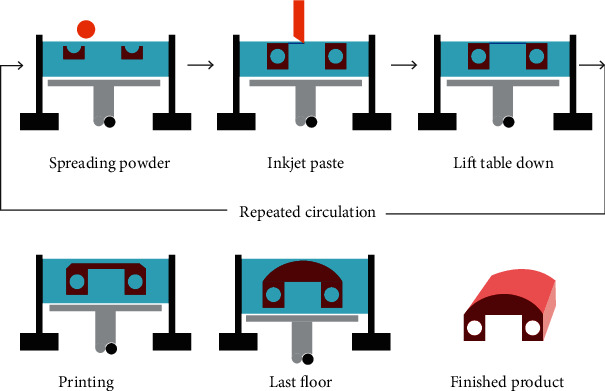
Three-Dimensional Printing.

In 1989, Emanuel M. Sach and John S. Haggerty of MIT applied for patents for 3D printing technology in the United States. After that, Emanuel M. Sachs and John S. Haggerty perfected the technology many times and eventually form a 3D printing process. The working principle of Three-Dimensional Printing (Figure 5): the powder material is laid flat in the working groove. Under the control of the computer, the nozzle sprays selectively based on the information of the cut workpiece, so that part of the powder is bonded and gradually forms a cross section. After the section is printed, the workbench drops a certain height, and then, the next layer is bonded until the workpiece is completely formed. The prototype bonded with a binder is of low strength and should be placed in a heating furnace for further curing or sintering. Cheekbones, mandibles, and skulls can all be printed by this technology.

The Three-Dimensional Printing process can process a wide variety of materials and has a fast forming speed, which can be used to print workpieces with complex structures. However, because it is only bonded by adhesive, the precision and surface quality of the printed workpiece are poor, and the strength of the prototype part is low, which requires subsequent treatment [16–18]. Due to its low cost, small size, fast molding speed, color printing can be realized and other advantages, this technology is getting more and more attention [19, 20].

In the 3D printing process, it is not easy to achieve both the large size and high precision of the formed part. At present, the working platform of powder spreading equipment on the market is generally not large. The main reason is that after the beam passes through the galvanometer, it can only precisely control the spot with uniform distribution of energy density in a certain area. So, how to improve the accuracy of optical components or realize multiple beams synchronous control is a development direction. In addition, 3D printing manufacturing is different from the general coating technology. It is coating on top of coating, which can be called “re-coating technology.” The thickness, flatness of each layer, and the degree of layer-to-layer bonding directly affect the stability and accuracy of the formed part, which need to be improved by adjusting equipment and process parameters.

The above manufacturing processes are all printed layer by layer; free-style 3D printing technology not only overcomes the structural design limitations of traditional layer-by-layer printing but also helps to achieve 3D printing of low-viscosity or slow-curing materials. As an emerging printing method, development free-style 3D printing system represents a major advance in biomedical engineering, because most of the materials used in the biomedical field involve soft substances, such as biomaterials, living cells, biopolymers, and silicones; these materials were previously printed using traditional AM systems. The free-style 3D printing system enables researchers to print these low viscosity materials and explore a wider range of soft materials for various applications. With the growing popularity of free-style 3D printing systems, a growing number of publications in the field of tissue engineering, vascularization, microaids, 4D printing, and manufacturing of elastomer. As an emerging printing method, free-style 3D printing system has been developed and solves many biomedical applications unmet need for producing, in particular, of low-viscosity material or slow-curing requirements.

## 3. 3D Printing Materials for Implantable Medical Devices

The development of 3D printing implantable medical devices is closely related to the development of medical level and material science, especially the development of biomaterials. Biological materials are substances that can be used to diagnose, treat, repair, or replace tissues and organs in the body or enhance their functions. They can be natural, artificial, or a combination of both.

Biomaterials used in 3D printing implantable medical devices can be divided into biomedical polymer materials, biomedical metal materials, biomedical ceramic materials, biomedical composite materials, and derived materials according to their properties.

### 3.1. Biomedical Polymer Materials.

Biomedical polymer materials are the earliest and most widely used materials in the field of biobiomedicine, and they have applications in all fields of medicine. Biomedical polymer materials can be divided into two categories: nondegradable and degradable on the basis of their properties. Nondegradable polymer materials include polyethylene, polypropylene [21], and polyformaldehyde. This type of material has good physical and mechanical properties, also can remain stable for a long time in a biological environment. Degradable biological materials include collagen [22, 23], cellulose [24], and chitin. The material is degraded under the action of the biological environment, and part of the degraded product is absorbed and partly eliminated with the normal metabolism of the human body. Biomedical polymer materials are widely used in the repair of cardiovascular stents, soft tissues, and hard tissues.

### 3.2. Biomedical Metal Materials

Biomedical metal materials refer to metals or alloys used in biomaterials, also known as metal materials for surgical implants. The clinically used biomedical metal materials mainly include stainless steel [25], titanium and titanium alloys [26, 27], cobalt-chromium-molybdenum alloys [28], and medical precious metals [29]. This type of material has high fatigue resistance and mechanical strength, good mechanical properties, corrosion resistance, and biocompatibility. It is widely used in bone and joint substitutes, spinal implants, cardiovascular implants, etc.

### 3.3. Biomedical Ceramic Materials

Biomedical ceramics have also become biomedical inorganic nonmetallic materials. They began as biomedical materials in the early eighteenth century and were clinically applied in China in the 1970s. Biomedical ceramics are divided into biologically inert ceramics, biologically active ceramics, and biodegradable ceramics. Bioinert ceramics include alumina, zirconia, and carbon. These materials have stable structure and high strength, good abrasion resistance, and stable chemical properties. Bioactive ceramics include bioglass [30, 31] and tricalcium phosphate [32, 33], which form strong chemical bonds with tissues through chemical reactions in the body. Biodegradable bioceramics can induce the growth of new bone since they can be absorbed in the living body, such as *β*-tricalcium phosphate bioceramics. Biomedical ceramics can be used to make artificial hip joints, artificial bones, valves, etc. The main problem of biomedical ceramics in clinical applications is poor strength and toughness.

### 3.4. Biomedical Composite Materials

Biomedical composite materials are composed of two or more different materials [34, 35]. Composite materials improve the performance of a certain material to a large extent, because it is a composite of different materials; the composite material will have new and unique properties that are different from the constituent materials. This new material property may be beneficial to the human body, or it may not be beneficial to the human body. Therefore, the composite material must not only have the expected physical and chemical properties but also must meet the requirements of biomechanical properties and biocompatibility. Biomedical composite materials include bone cement, coating materials, and nano-phosphoric lime. The biomedical composite material is widely used in repairing or replacing human tissues and organs or enhancing their functions and the manufacturing of artificial organs [36–38].

### 3.5. Derived Materials

Bioderived materials are also called biorenewable materials, which are formed from natural biological tissues through special processing. Since biological tissues have lost their vitality after special treatment, biologically derived materials are inanimate materials. Bioderived materials have similar configurations and functions to natural tissues, or because they have a composition similar to natural tissues, bioderived materials play a major role in the maintenance and replacement of human dynamic processes [39]. The biological material is widely used in artificial heart valves, skin masks, bone restorations, vascular restorations, etc.

For the moment, research on biomedical 3D printing technology is springing up. Many achievements have been made in medical 3D printing materials, especially in tissue engineering scaffold materials. However, biomedical 3D printing technology and its materials are still an emerging field, and various researches are still in the initial stage. To truly realize the clinical application of a long distance, there are still great challenges. Mechanical metamaterials such as superhard materials, auxiliary materials, hyperelastic materials, and self-assembled and programmable materials can be used in cartilage tissue engineering, bone tissue engineering, skin tissue engineering, vascularized tissue engineering, etc. Metamaterials begin with porous implants; these include biomimetic materials created from naturally derived buildings. Mechanical metamaterials also provide radical designs for functional tissue scaffolds; it has shown promising potential in bone tissue regeneration and orthopedic implants, with extreme rigid weight ratio, tunable hydraulic penetration, and even higher surface volume ratios; intelligent materials are a class of outstanding engineering materials due to their shape memory effect, Shape Memorty Materials (SMM) generally classified as Shape-Memory Polymers (SMP), Shape-Memory Ceramics (SMC), Shape Memory Alloys (SMA), and Shape-Memory Hydrogels (SMHs); out of which, SMP became a more notable and researchable class, intelligent materials to further open up the possibility more bone regeneration.

## 4. Typical 3D Printed Implantable Medical Devices

3D printed implantable medical devices are applied to various parts of the human body. Typical products include vascular stents, heart valve prostheses, orthopedic implants, and artificial joint prostheses.

### 4.1. Vascular Stents

**Figure 6:**
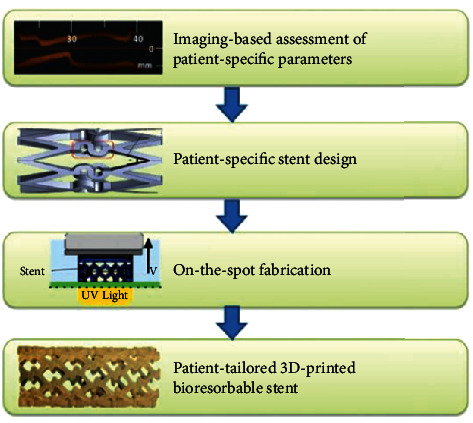
Process of preparing blood vessel stent by 3D printing technology [42].

Cardiovascular disease (CAD) is a disease that affects the myocardium, heart valves, or blood vessels, and its morbidity and mortality are gradually increasing. Around the world, cardiovascular disease kills a large number of people every year, and it is called the number one killer of health [40]. The treatment of cardiovascular disease has become the focus of research by relevant scholars in various countries. The methods of treating cardiovascular diseases include drug treatment, external surgical treatment, and vascular stent interventional treatment [41–43]. The effect of drug treatment is not obvious, the recovery period after external surgery is long, and both of them may cause the patient to suffer secondary injury. As a minimally invasive surgery, vascular stents cause less pain, short operation time, less trauma, and quicker recovery after surgery. Therefore, vascular stent interventional therapy has become the main method for the treatment of cardiovascular diseases.  Figure 6 is the process of preparing blood vessel stent by 3D printing technology.

**Figure 7:**
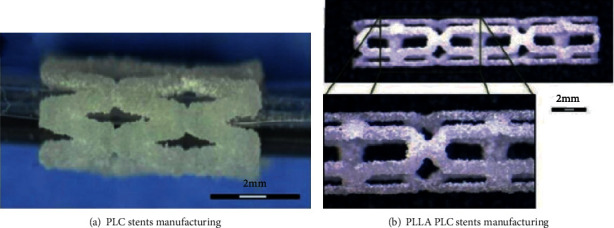
Vascular stent printed by SLM technology [44].

Flege et al. [44] made use of selective laser melting (SLM) 3D printing technology to prepare the biodegradable vascular scaffolds for PLLA and PCL powder particles and then made the surface of the stent smooth by dip coating and spray treatment. The biodegradable stent is shown in Figure 7. The experimental results show that the method and the bioabsorbable scaffold prepared by the materials have good biocompatibility. Figure 7 is the vascular stent printed by SLM technology.

**Figure 8:**
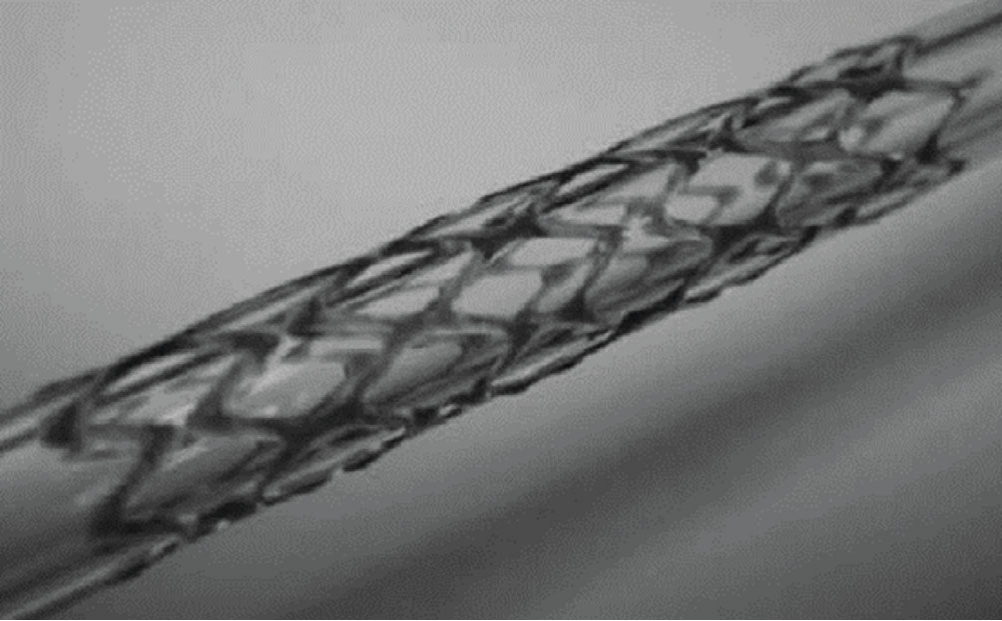
Biodegradable vascular stents prepared by RSF system [45].

Kaesemeyer et al. [45] indicated the biodegradable vascular stent fabrication by the rapid vascular stent fabrication (RSF) system shown in Figure 8. The vascular stent is prepared from a copolymer of lactide, glycolide, *ε*-caprolactone, and lovastatin. The vascular stent not only has a supporting function but also releases drugs in the body that can be used to treat damaged endothelium and prevent thrombosis in the stent. Figure 8 shows the biodegradable stent prepared by RSF system.

**Figure 9:**
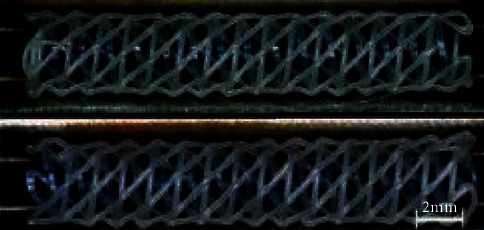
PCL spiral vascular stent [46].

Park et al. [46] prepared a spiral bioabsorbable PCL vascular stent (Figure 9). The drug coating of PLGA and PEG mixed with sirolimus was prepared by ultrasonic atomization and then implanted in the body for experiments. The release kinetics of Rolimus in the stent is a continuous curve, and the vascular intimal hyperplasia is reduced.

Van Lith et al. [47] used the 3D printing technology of microcontinuous liquid interface production to print a kind of curable biomaterial based on bioabsorbable citrate and prepared a bioabsorbable vascular stent with rapid, high-resolution, and customized design. Misra et al. used 3D printing technology to print the composite material doped with biodegradable polymer PCL and graphene nanoflakes, then prepared a vascular scaffold which can realize dual drug release.

From the domestic and foreign research status, 3D printing has a bright future in the application of vascular stent. However, the application of 3D printing technology in vascular stents is still in its initial state, and its application is still immature. At present, there are some problems in the application of 3D printing technology in vascular stents, such as vascular scaffolds made of biodegradable polymeric materials are deficient in mechanical properties due to the influence of material properties, and the positioning accuracy of vascular scaffolds is poor due to their expansion, which will affect the subsequent treatment.

### 4.2. Prosthetic Valve

**Figure 10:**
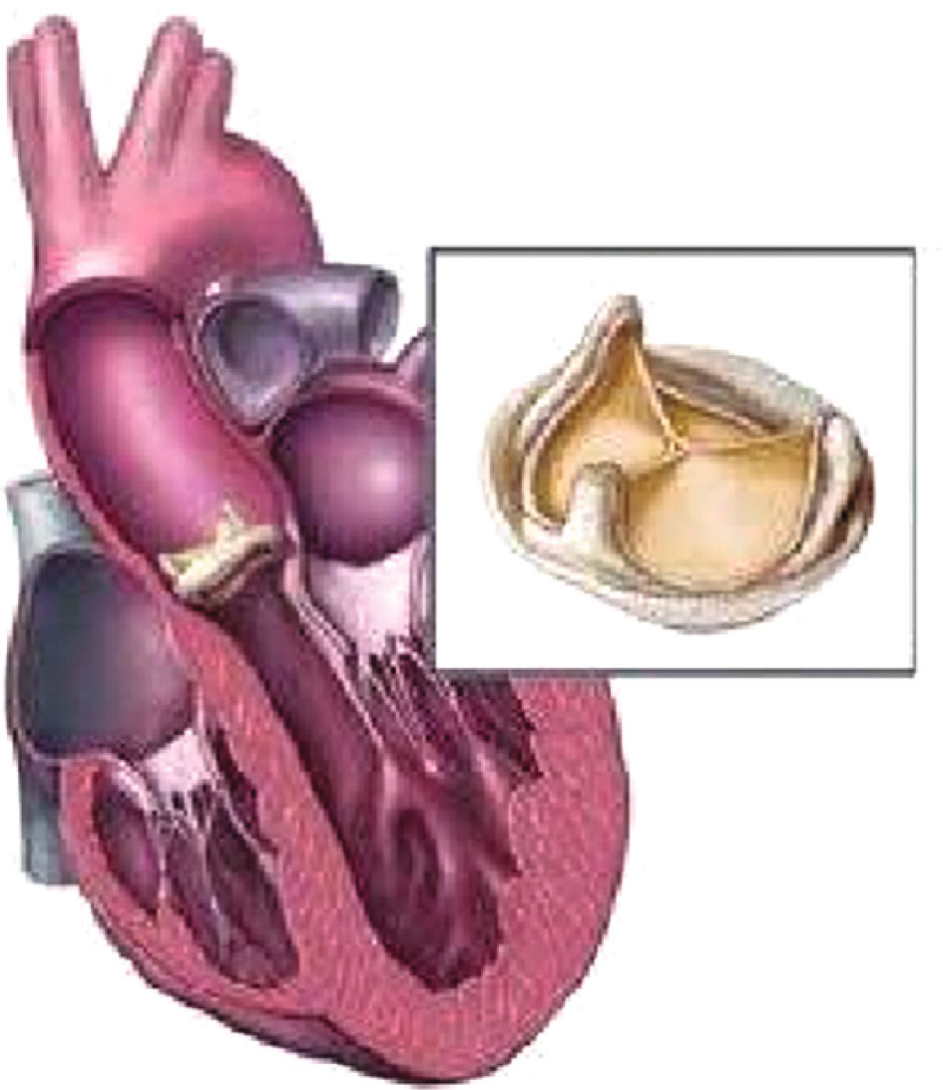
Biological heart valve replacement [50].

**Figure 11:**
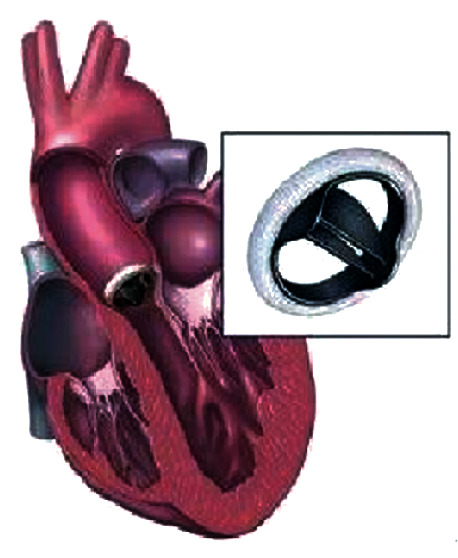
Mechanical heart valve replacement [50].

The function of heart valves is to ensure that the blood in the heart flows in the correct direction. Heart valve disease will endanger human health and affect the normal quality of life of humans. Myocardial infarction and senile valve disease due to aging are common in the elderly [48]. Hyperlipidemia, hypertension, and chronic kidney disease are common in modern adults, which can cause heart valve damage. Heart valve disease is a common heart disease in modern medicine. Valve lesions can hinder the normal flow of the blood and increase the burden of the heart, thus causing damage to heart function and leading to heart failure. There are three methods to treat heart valve disease: drug treatment, external surgical treatment, and interventional treatment. External surgical treatment refers to the use of artificial heart valve replacement or valvuloplasty for treatment, which is an effective treatment for high-risk patients, and it is also a radical cure for heart valve disease. The 3D printed heart valve can be customized based on different patients, so as to improve the accuracy and stability, reduce the rejection reaction of the patient's body, and improve the success rate of heart valve replacement [49]. Biological heart valve replacement and mechanical valve replacement are shown in Figures 10 and 11.

The development of artificial heart valves has gone through the stages of mechanical valves, biological valves, interventional valves, and tissue-engineered valves [51, 52]. Tissue valve is an active heart valve constructed by 3D printing technology.

**Figure 12:**
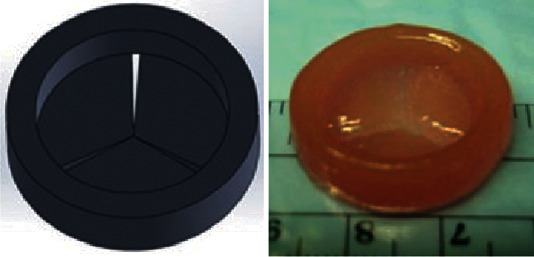
Aortic valve model and printed product [54].

In the 1960s, China began to study artificial heart valves. Professor Yongzhi Cai successfully performed mitral valve replacement with domestic cage valve [53]; the biomaterial artificial heart valve developed by the Beijing Fuwai Hospital has been successfully used in aortic valve replacement surgery; Kapetanovic et al. [54] used hyaluronic acid and methacrylate composite hydrogel to load human aortic valve interstitial cells, then increased the viscosity of the hydrogel by increasing the concentration of methacrylate, thereby promoting the transmission of information between cells and maintaining human fiber cell phenotype (Figure 12) and aortic valve model and printed entity.

There are still some deficiencies in the preparation of aortic valve in the world. There are some differences between the designed aortic valve and the natural aortic valve, and the non-Newtonian characteristics of the hydrogel are ignored in the printing process of the aortic valve. The phenomenon of swell will appear from the microneedle extrusion, which will affect the surface accuracy and dimensional accuracy of the aortic valve.

### 4.3. Orthopedic Implants

Bone tissue is an important part of the human body, as well as an important part of the bones. It plays an irreplaceable role in the human body. Bone tissue has a strong ability to regenerate bone and can repair and heal itself. However, when the bone defect reaches a certain degree, the bone tissue corresponding to the bone defect will not be able to repair itself [55, 56], and there will be some images in the defect site nonbone tissues like fibers grow; clinically, there are more and more bone defects caused by trauma, tumor resection, accidents, etc., so the demand for bone implants is increasing.

Methods for repairing bone defects include autografts, allografts [57, 58], and artificial bone substitute materials such as metal and polymer materials. Autologous bone transplantation requires two operations. The patient suffers a lot of pain, and the human body has a limited amount of autologous bone, which cannot provide unlimited bone sources [59]. The operations are not all successful [60]. However, due to different constitutions, allograft may cause immune rejection and carry the risk of infectious diseases from the donor [61]. Therefore, the research of artificial bone repair has become the focus of many related scholars [62].

Artificial bone scaffolds prepared by traditional methods such as gas foaming [63, 64], fiber bonding, freeze-drying, phase separation [65], and particle leaching cannot precisely control the cell pore shape and pore size of the artificial bone scaffold, which makes the artificial bone scaffold prepared biologically the performance does not meet the demand well. In recent years, 3D printing technology to manufacture artificial bone scaffold has become a preferred scheme, which has great potential in drilling.

In March 2014, the First Affiliated Hospital of the Fourth Military Medical University of Chinese PLA (Xijing Hospital) implemented 3D printing titanium alloy shoulder blade prosthesis, clavicular prosthesis, and pelvic prosthesis for three patients with bone tumors. The first two cases were the first in the world, and the last one was the first in Asia, which made a significant step in the application of 3D printing technology in bone tissue engineering [66]. Saijo et al. used 3D printing technology to prepare tricalcium phosphate powder into a personalized prosthesis and achieved satisfactory results in clinical applications; Dai Kerong and others used 3D printing technology to prepare metal materials into artificial pelvis, then successfully completed the replacement of the artificial pelvis and achieved satisfactory results in clinical practice. The titanium alloy clavicle and scapula prepared by Pei Yanjun and others using 3D printing technology were successfully implanted into the body of bone tumor patients, and the clinical effect was good.

With the development of society, 3D printing will be more and more widely used in orthopedics. However, the application of 3D printing technology in orthopedics is at the initial stage, and the technical materials are immature; there are still some problems: the 3D printed bone implant lacks large sample data and follow-up data; due to the high clinical requirements for implant materials, there are still some problems in material selection; the structure of human tissues and organs is complex, and the accuracy of printing still needs to be improved.

### 4.4. Artificial Joint Prostheses

Osteoarthritis (OA) is also known as degenerative arthritis. The disease damages articular cartilage and eventually leads to degenerative joint disease of the whole joint, which can lead to loss of normal joint movement and joint dysfunction. Joint replacement surgery, as a more difficult operation in the field of orthopedics, is used in clinical treatment of joint dysfunction, thereby restoring the shape and function of the joint.

Total hip arthroplasty (THA) is an operation in which artificial hip prosthesis is used to replace the dysfunctional hip joint [67, 68]. Hip joint prosthesis is suitable for the elderly with fresh or old femoral neck fractures and nonunion; middle-aged and elderly patients with severe degenerative osteoarthritis and avascular necrosis of the femoral head. Clinically, Cheng Wenjun and others performed artificial total hip replacement surgery for patients with femoral neck fractures, primary hip joints, and secondary osteoarthritis, which used traditional prostheses and 3D printed titanium alloy bones. There are two methods of trabecular metal cups. After the operation, it was found that the hip joint movement function of the two groups was greatly improved. However, the initial stability of the prosthesis of the 3D printed titanium alloy trabecular metal cups was better, and the initial stability of the bone in growth is better.

**Figure 13:**
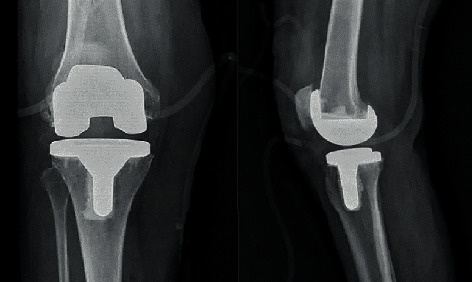
Total knee replacement X-ray [70].

Total knee arthroplasty (TKA) is the most effective surgery for treating moderate to severe knee arthritis [69]. Knee joint prostheses refer to surgical implants used to replace the articular surfaces of the femur and tibia on both sides of the knee. The X-ray films of total knee arthroplasty were as shown in Figure 13.

Finger joint trauma is a common orthopedic disease in clinic. The important methods for the treatment of finger joint dysfunction are joint transplantation, arthroplasty, joint fusion, and artificial joint replacement. Artificial joint replacement is widely accepted for its advantages of finger function reconstruction and hand knuckle stability restoration. In 1940, Burman [71] carried out the alloy metacarpal head hemi-joint replacement technique, which is regarded as the prototype of the hand facet joint replacement technique. In 1959, the clinical application of total joint replacement prosthesis designed by Brannon and Klein [72] led to the widespread development of artificial joint replacement. In 1962, Swanson and Peltier [73] proposed a new concept of artificial finger joint prosthesis. In 1969, Niebauer et al. [74] and ChiaRi and Trieb [75] made improvements on the basis of Swanson. In the 1970s, the design of the prosthesis was more in line with the biomechanics and the application of the prosthesis that was close to the normal joint anatomy. At present, there are many types of human manual articular surface replacement prostheses used in clinical practice, including Linscheid et al. [76].

At present, 3D printing technology has been widely used in joint replacement surgery, but the innovation of the material and process of the prosthesis still needs to be enhanced to improve the stability and effectiveness of the prosthesis in the body.

### 4.5. Human Organs

3D printing technology has a wide range of applications in the construction of functional tissue, including kidney, liver, multilayer skin [77], and other tissues or organs that can replace damaged or diseased tissues or organs.

Organ transplantation is the best treatment for end-stage renal disease (ESRD). However, the number of existing kidney donors is far less than the number of kidneys needed. For this reason, the use of 3D printing technology to print artificial kidneys has emerged to satisfy the donors required for surgery. Organovo used 3D printing technology to prepare the first whole-cell kidney tissue. King et al. [78] used 3D printing technology to prepare an in vitro model of three-dimensional proximal tubule tissue, which ushered in the dawn of organ transplantation and kidney regeneration. In 2011, Anthony Atala [79] of the Wake Forest University showed people 3D printing kidney technology; so far, 3D printing artificial organ technology has made great progress. The 3D printing technology of kidney organs is aimed at printing organs and tissues with organ functions. With the development of 3D printing technology and the continuous progress in the field of material science, it is expected that 3D printing artificial kidney can meet the needs of human body, improve the situation of kidney shortage, reduce people's pain, and improve people's life.

As one of the indispensable organs of human body, the structure of liver is complex. Compared with other organs, the liver has strong regeneration ability. But due to the small number of donors and the long regeneration cycle of its own liver, 3D printing of liver organs has become the core solution to solve the problem. In 2013, ZenI et al. [80] printed a translucent human liver model. Alkhouri and Zein [81] explored the use of 3D bioprinting technology to create miniature liver, which could be used to replace the pathological liver of end-stage liver failure. In 2013, Organovo printed a miniature liver with most functions of natural liver.

Skin transplantation is necessary for facial burns. If the burn area is too large, the amount of skin grafts required will increase, so that the supply exceeds demand. Using 3D printing technology to create artificial skin can not only meet the needs of skin transplantation but also reduce the pain of patients. Koch et al. [82] used 3D printing technology to print collagen, keratinocytes, and fibroblasts to prepare skin analogues. In 2013, Koch et al. [83] used 3D printing technology to print biological materials and prepared human skin tissues, which provided a reliable source for skin transplantation. Lee et al. [84] used 3D printing technology to print collagen, keratinocytes, and fibroblasts, respectively, to prepare skin tissue structure.

The emergence of 3D printing technology has given a solution to the problems faced by organ transplantation. But at present, 3D printing still needs to improve the resolution of 3D printing technology and the flexibility of biomaterials in the aspect of printing organs, so as to realize more complex and composite tissue or organ structures for clinical applications.

In general, with the development of materials science, technology, and imaging, 3D printing technology will be more and more widely used in cardiovascular and orthopedic fields. New 3D printing technologies such as in vivo direct printing, personalized generated heart implant device, and orthopedic implants will also greatly enrich the connotation of 3D printing technology and better benefit the cardiovascular patients and bone defect patients. But at present, the problems and challenges of 3D printing technology are also prominent. The first is the accuracy of printing, which involves imaging mode and printing mode. The second is the material problem; it is a long-term task to find materials that meet the functional requirements, biological requirements, economic requirements, and technological requirements. The third problem is vascularization, the ability of 3D-printed tissues and organs to survive and function in the long term only based on stable vascularization. It is believed that 3D printing technology in cardiovascular and orthopedic applications will shine.

## 5. Conclusions

As a new type of digital printing technology, 3D printing has many advantages and huge applications in medicine, which makes every country invest more energy in the research and development of it. 3D printing technology has been applied in various fields of clinical medicine, especially in the application of implantable medical devices, which has been paid more attention by relevant scholars in various countries. Although 3D printing technology is currently in full swing, its technology still faces many challenges. For example, one of its challenges is how to develop various 3D printing raw materials. The second challenge is that 3D printing lacks industry standards for implantable medical devices. The third challenge is that the product technology chain has not yet been fully formed. As for the progress and development of 3D printing technology, the research on materials is the key. The biomaterials in the future should have the best mechanical properties, diffusion coefficient and biocompatibility, which are the basis and direction for 3D printing technology to advance in the future medical field. In addition, if the combination of biomaterials and printing technology can solve the problem of combining different types and functions of cells into a three-dimensional structure with complex functions and realize their basic functions. This technology will usher in a revolutionary change in 3D printing technology and biological tissue engineering, and the repair of human tissues and the transplantation of tissues and organs will be completely met by biological printing technology. In the future, independent 3D printing manufacturing centers for human organs can be built. This center can make corresponding implants in time according to the needs of the disease, so that the complex diseases encountered in clinical practice can be quickly solved, and patients' pain can be timely and effectively controlled.

The in situ 3D printing proposed for 3D printing is the implantation of original tissues in original body parts, avoiding foreign graft rejection and infection, for better recovery of the patient's body, and compared to bioprinted implants, in situ 3D printing is more accurate in size and shape, and it also overcomes the shortcomings of difficult implantation. One of the remaining challenges at the core of the further development of 3D printing in situ is the optimization and crosslinking of inks used in the process, Agostinacchio et al. [85] proposed the use of silk fibroin as an ink printing formula; different formulations of silk-based inks should be studied, characterized, and standardized, get shape fidelity, and at the same time, avoid the possible in vivo cytotoxic effects of photoinitiators during photopolymerization and expand the scale of manufacturing to be used in clinical practice. The emergence of smart materials or programmable materials, which can be converted by external stimuli, provides exciting new opportunities for 3D printing technology. This combination has led to a new field called 4D printing; the scale of 4D bioprinting to print bones has fascinating prospects. Compared with 3D printing, 5D printing saves 25% of materials in the printing process. 5D printing is a new branch of additive manufacturing. In this technology, the print head and the printable object have five degrees of freedom. Instead of the flat layer, it produces curved layers. In this process, the print part moves while the printer head is printing. So, printing undertakes the curve path of the part being printed rather than moving through a straight layer as in the case of 3D printers. The main advantage of this technology is to create a part with a curved layer with improved strength. A 5D printed model provides potential to fabricate artificial bone for surgery. Because human bones are not flat and having a curved surface, there is a requirement to manufacture artificial bones with 5D printing to provide excellent strength to these bone implants. This technology has great potential to fulfill this primary requirement. As the future of medical industry, 3D printing will become an important means of precision medicine and personalized medicine in the future. In the future, multidimensional printing will become a bright spot and vane in the medical field in the future and will promote the development of medical biology research in a country.
